# Intrinsic capacity and comorbidity burden capture distinct but age-driven dimensions of health in older postmenopausal women

**DOI:** 10.1007/s40520-026-03456-7

**Published:** 2026-07-11

**Authors:** Oliveri Cecilia, Gaudio Agostino, Soraci Luca, Cesari Matteo, Catalano Antonino

**Affiliations:** 1 Department of Clinical and Experimental Medicine, University Hospital of Messina, Messina, Italy; 2 Department of Clinical and Experimental Medicine, University of Catania, Catania, Italy; 3 Unit of Geriatric Medicine, Italian National Research Center on Aging (IRCCS INRCA), Cosenza, 87100 Italy; 4 Department of Clinical Science and Community Health, University of Milan, Milan, Italy

**Keywords:** Intrinsic capacity, Comorbidity, Ageing, Functional reserve, ICOPE, Older adults

## Abstract

**Background:**

Intrinsic capacity (IC) and comorbidity burden are both associated with health outcomes in older postmenopausal women, yet whether they capture independent dimensions of health remains unclear.

**Aims:**

To examine whether comorbidity burden is independently associated with IC in older postmenopausal women, and to evaluate the role of age as a potential confounder of this relationship.

**Methods:**

IC was evaluated across five WHO-defined domains using ICOPE-aligned tools, and the number of preserved IC components (range 0–6) was calculated as an index of functional reserve. Comorbidity burden was quantified with the Charlson Comorbidity Index (CCI).

**Results:**

A total of 200 postmenopausal women were included (mean age 68.2 ± 8.5 years, range 46–89). The mean CCI score was 3.5 ± 1.9 and the mean number of preserved IC components was 3.4 ± 1.3. Preserved IC components were inversely correlated with CCI (ρ = −0.33, *p* < 0.001). In multivariable analysis, CCI was not independently associated with preserved IC (β = −0.036, 95% CI − 0.142 to 0.070, *p* = 0.505), whereas age was a strong independent predictor (β = −0.078, 95% CI − 0.100 to − 0.055, *p* < 0.001). The partial correlation between IC and CCI controlling for age was negligible (*r* = 0.003, *p* = 0.962). Sensitivity analyses using Poisson regression and a 5-component IC score excluding the vision domain confirmed these findings. CCI was not independently associated with any individual IC domain in domain-specific logistic regression models.

**Discussion:**

The observed association between IC and comorbidity burden was largely explained by age, suggesting that these constructs capture overlapping but not identical dimensions of health vulnerability.

**Conclusions:**

IC captures dimensions of functional vulnerability not fully accounted for by comorbidity burden alone.

**Supplementary Information:**

The online version contains supplementary material available at https://doi.org/10.1007/s40520-026-03456-7.

## Introduction

The global ageing of populations has brought two complementary paradigms to the forefront of geriatric medicine: a disease centred model, focused on the diagnosis and treatment of specific conditions, and a functional capacity model, which prioritises the preservation of physical and mental reserves across the life course. Multimorbidity - commonly defined as the coexistence of two or more chronic conditions - affects more than one third of adults worldwide and over 60% of older adults in Europe, and is a major driver of functional decline, health-care utilisation, and mortality [1, 2].

The World Health Organization (WHO) has defined intrinsic capacity (IC) as the composite of all physical and mental capabilities that an individual can draw upon at any given time, encompassing five domains: cognition, locomotion, vitality, psychological well-being, and sensory function (vision and hearing) [3, 4]. IC is operationalised in clinical practice through the Integrated Care for Older People (ICOPE) framework, which is designed to detect and address declines in functional reserves at the community level [5]. A growing body of evidence from longitudinal studies and systematic reviews indicates that IC impairment independently predicts functional decline, disability, and mortality in older adults [6, 7]. Recently, Chen et al. validated the IC construct in a large population of over 60,000 older adults across 15 European countries; interestingly, age was the main predictor of IC decline over time, but a substantial gender gap emerged, especially in Southern Europe: indeed, women scored systematically lower than men across most IC domains [8]. This gap can have more important clinical consequences, especially among women, given that the menopausal transition can accelerate the decline in multiple IC domains through multiple biological mechanisms. For similar biological reasons, postmenopausal women constitute a population characterized by a substantial burden of comorbidities. The abrupt decline in estrogen levels at menopause leads to the loss of their cardioprotective, renoprotective, and osteoprotective effects, thereby accelerating the development of a broad spectrum of conditions, including osteoporosis, diabetes, cardiovascular diseases, and metabolic syndrome [9–14].

Although both IC and comorbidity burden are consistently associated with health-related outcomes, their interrelationship remains incompletely characterized. Conceptually, comorbidity reflects the cumulative burden of multiple chronic conditions, whereas IC denotes the overall functional reserve of the individual; both constructs are closely linked to the ageing process and may therefore represent partially overlapping dimensions of vulnerability [15].

Determining whether these constructs capture independent dimensions of health has clear clinical implications, particularly in postmenopausal women, among whom both impairment in IC and a high comorbidity burden are highly prevalent. Furthermore, a predominantly disease-centred approach may systematically underestimate the overall severity of health vulnerability.

In this context, we investigated whether comorbidity burden is independently associated with IC in older postmenopausal women and evaluated the extent to which this association is explained by chronological age. As a secondary aim, we investigated the potential contribution of reproductive factors - specifically years since menopause and reproductive lifespan, as proxies of cumulative oestrogen exposure - and socioeconomic characteristics, including educational attainment and occupational status, to IC, hypothesising that these factors may exert independent effects on functional reserves beyond those attributable to age alone.

## Methods

### Study design and participants

We conducted a cross-sectional study of postmenopausal women aged 50 years or older with a diagnosis of osteoporosis or osteopenia, consecutively evaluated at the Outpatient Clinic for Bone and Mineral Metabolism Disorders of the University Hospital of Messina, Italy. Women with severe cognitive impairment precluding informed consent, severe disability (bedridden status), acute medical conditions at enrolment, or receiving chronic corticosteroid therapy which may directly impair locomotion (via steroid myopathy), vitality, and psychological well-being, or other treatments likely to interfere with the IC assessment (e.g., antipsychotics, benzodiazepines, or opioids at high doses), as conceptualised within the WHO ICOPE framework, were excluded. The study protocol was approved by the local Ethics Committee (protocol 23/19), and all participants provided written informed consent prior to enrolment. All assessments were performed by a geriatric trainee physician specifically qualified in the administration of ICOPE-aligned assessment tools. In addition to IC and comorbidity assessment, sociodemographic data (education level and occupational status) and reproductive characteristics (age at menarche, age at menopause, reproductive lifespan defined as the interval between menarche and menopause, and years since menopause) were collected from all participants at enrolment.

### Assessment of intrinsic capacity

IC was assessed in accordance with the WHO ICOPE framework across its five core domains: cognition (Mini‑Cog), locomotion (Short Physical Performance Battery [SPPB]), vitality (Mini Nutritional Assessment [MNA]), psychological well-being (Patient Health Questionnaire‑9 [PHQ‑9]), and sensory capacity. Sensory capacity was operationalised as two distinct components—vision (WHO eye chart) and hearing (digits‑in‑noise test). Each domain was classified as preserved or impaired using established thresholds: Mini‑Cog score ≤ 2 for cognitive impairment; SPPB score ≤ 8 for impaired locomotion; MNA score < 24 for reduced vitality; PHQ‑9 score ≥ 5 for psychological impairment; and inability to read the 6/12 line on the WHO eye chart or a digits‑in‑noise score below the age‑adjusted cut‑off for sensory impairment. The total number of preserved IC components (range 0–6), with vision and hearing considered separately, was used as the primary outcome, reflecting overall IC and functional reserve.

### Comorbidity burden

Comorbidity burden was quantified with the Charlson Comorbidity Index (CCI; range 0–37), a validated instrument that assigns weighted scores to individual comorbid conditions according to their adjusted contribution to mortality risk [16]. In the present analyses, the CCI was modelled as a continuous variable.

### Statistical analysis

All analyses were performed using R software (version 4.3.1). As the distributions of the number of preserved IC components and CCI scores deviated from normality (Shapiro–Wilk test, *p* < 0.001 for both), non-parametric methods were applied for bivariate analyses. The association between preserved IC components and comorbidity burden was examined using Spearman’s rank correlation coefficient, and differences in CCI across strata of preserved IC components (range 0–6) were assessed using the Kruskal–Wallis test; effect size was estimated using eta-squared (η²). To evaluate whether comorbidity burden was independently associated with IC after accounting for age, a multivariable linear regression model was fitted with the number of preserved IC components as the dependent variable and CCI score and age as independent variables. Partial correlations between IC and comorbidity burden, controlling for age, were estimated using the pcor.test function from the ppcor package. Model performance was evaluated using the adjusted R² and F statistics. Additional exploratory analyses were undertaken to investigate the potential contribution of reproductive (years since menopause, reproductive lifespan) and socioeconomic factors (education level, occupational status) to IC. As pre-specified sensitivity analyses, a Poisson regression model was fitted given the count nature of the outcome, with results expressed as incidence rate ratios (IRR) with 95% confidence intervals; analyses were repeated using a 5-component IC score excluding the vision domain given its near-universal prevalence (94.5%); and domain-specific logistic regression models were fitted to examine the association between CCI and impairment in each individual IC domain, adjusting for age, with model fit assessed using the Nagelkerke pseudo-R². All statistical tests were two-tailed, and a p value of less than 0.05 was considered statistically significant.

## Results

The study population comprised 200 postmenopausal women with a mean age of 68.2 years (SD 8.5). The mean CCI score was 3.5 (SD 1.9), indicating a substantial comorbidity burden consistent with a tertiary outpatient setting (Table 1). Impairment in IC domains was highly prevalent: only five participants (2.5%) showed no deficits across any IC component. Sensory capacity was the most frequently affected domain, with visual impairment observed in 189 participants (94.5%) and hearing impairment in 81 (40.5%). Psychological impairment was present in 95 participants (47.5%), followed by impaired locomotion (37.5%), cognitive impairment (25.0%), and reduced vitality reflected by risk of malnutrition (12.5%) (Supplementary Table 1).

**Table 1:** Baseline clinical characteristics and prevalence of preserved IC components in the study population (*n* = 200, values are mean ± SD unless otherwise specified)

	Participants (n = 200)
Age (yr)	68.2 ± 8.5
Weight (kg)	62.9 ± 12
Height (cm)	155.8 ± 6.1
BMI (kg/m^2^)	25.9 ± 4.9
Reproductive characteristics	
- Age at menarche (yr)	12.4 ± 1.2
- Age at menopause (yr)	48.4 ± 5.2
- Reproductive lifespan (yr)	36 ± 5.2
- Years since menopause (yr)	19.9 ± 8.8
Socioeconomic characteristics	
Education level [n (%)]	
- Primary school	40 (20)
- Middle school	92 (46)
- High school	44 (22)
- University degree	24 (12)
Occupational status	
- Housewife	90 (45)
- Employed	71 (35.5)
- Unemployed	5 (2.5)
- Retired	34 (17)
Participants with preserved IC components	
- Locomotor [n (%)]	125 (62.5)
- Vitality [n (%)]	175 (87.5)
- Visual [n (%)]	11 (5.5)
- Hearing [n (%)]	119 (59.5)
- Psychological [n (%)]	105 (52.5)
- Cognitive [n (%)]	150 (75)
Charlson Comorbidity Index	3.5 ± 1.9

Abbreviations: BMI, body mass index; IC, intrinsic capacity; SD, standard deviation

**Fig. 1:**
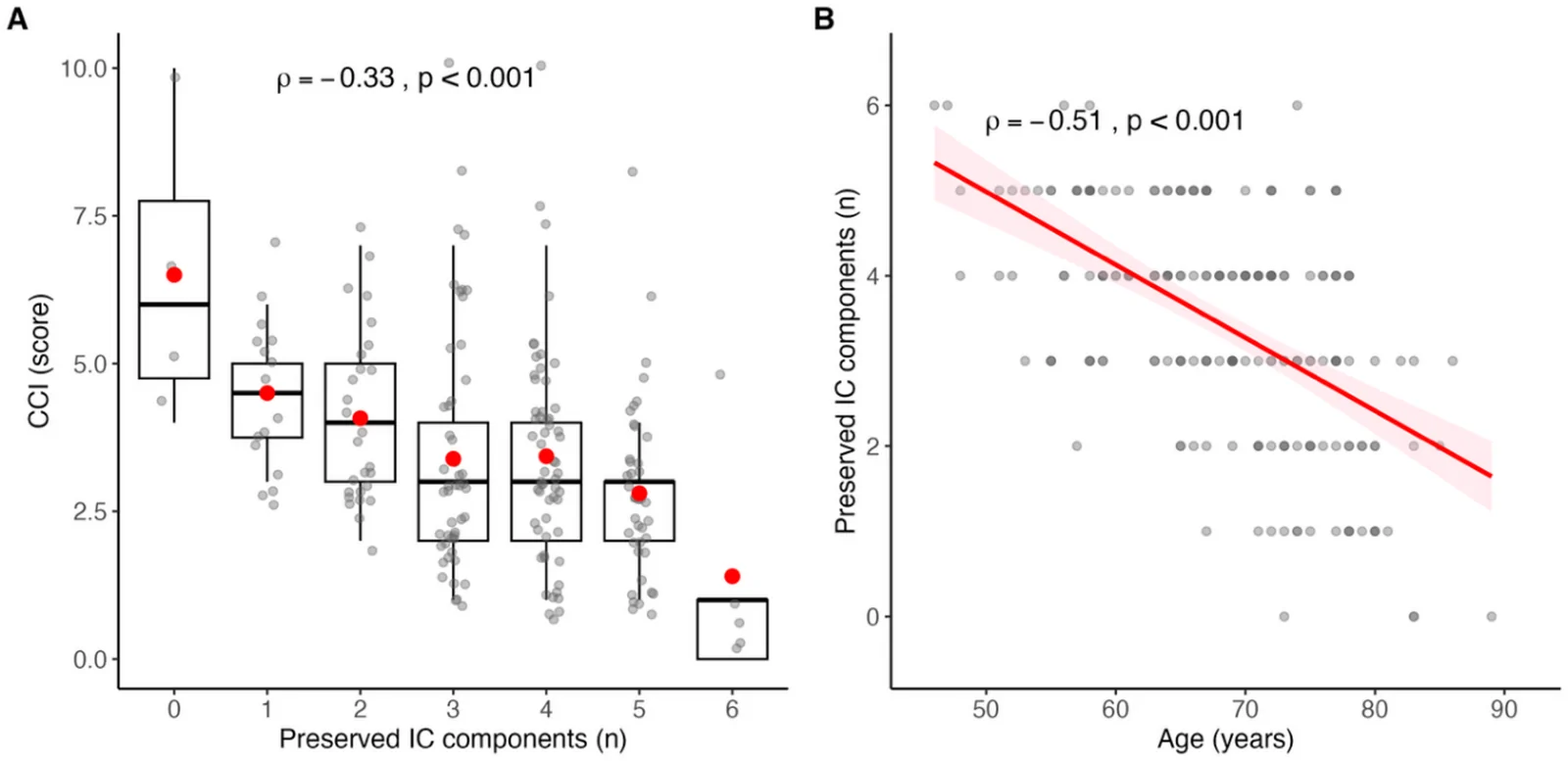
Relationship between intrinsic capacity, comorbidity burden, and age. (**A**) Distribution of Charlson Comorbidity Index (CCI) scores across strata of preserved intrinsic capacity (IC) components. Boxplots represent median and interquartile range, with individual data points overlaid and mean values indicated by red dots. (**B**) Association between age and number of preserved IC components. The fitted regression line with 95% confidence interval shows a significant inverse relationship

The zero-order correlation of preserved IC with CCI was significant (Spearman’s ρ = −0.33, *p* < 0.001) but was attenuated by 99% after controlling for age (partial *r* = 0.003, *p* = 0.962), indicating that the crude association was entirely attributable to the shared variance of both CCI and IC with age. Preserved IC was strongly correlated with age (Spearman’s ρ = −0.51; *p* < 0.001), and comorbidity burden was itself associated with age (Spearman’s ρ = +0.66; *p* < 0.001). CCI scores differed significantly across strata of preserved IC components (Kruskal–Wallis χ² = 31.66, df = 6, *p* < 0.001, η² = 0.133), with higher levels of preserved IC generally associated with lower comorbidity burden, although substantial overlap across strata was observed (Fig. 1A).

In multivariable linear regression, CCI score was not independently associated with IC after adjustment for age (β = −0.036, 95% CI − 0.142 to 0.070; *p* = 0.505) (Table 2). Age was a strong and independent predictor of IC (β = −0.078, 95% CI − 0.100 to − 0.055; *p* < 0.001), corresponding to a reduction of approximately 0.78 preserved IC components — roughly one domain lost — per 10-year increment in age. The model explained 28.0% of the variance in preserved IC components (adjusted R² = 0.280; F(2,197) = 39.8; *p* < 0.001). Exploratory analyses incorporating socioeconomic and reproductive variables revealed no independent associations with IC after adjustment for age (Supplementary Table 2).

**Table 2:** Multivariable linear regression model for preserved intrinsic capacity as dependent variable

Variables	β	SE	95% CI	*p*-value
CCI	-0.036	0.054	-0.142 to 0.070	0.505
Age (yr)	-0.078	0.012	-0.100 to -0.055	< 0.001

Adjusted R² = 0.280; F(2,197) = 39.8, *p* < 0.001. CCI, Charlson Comorbidity Index; SE, standard error; CI, confidence interval

Sensitivity analyses confirmed the robustness of these findings. In the Poisson regression model, CCI was not independently associated with preserved IC (IRR = 0.989, 95% CI 0.937–1.040, *p* = 0.669), whereas age remained a significant predictor (IRR = 0.978, 95% CI 0.968–0.989, *p* < 0.001). When analyses were repeated using a 5-component IC score excluding the vision domain, results were consistent (linear: CCI β = −0.036, 95% CI − 0.141 to 0.069, *p* = 0.502; Poisson: CCI IRR = 0.989, 95% CI 0.937–1.041, *p* = 0.669; age β = −0.071, *p* < 0.001; age IRR = 0.980, *p* < 0.001). In domain-specific logistic regression models adjusted for age, CCI was not independently associated with impairment in any individual IC domain (all *p* > 0.05). Age was significantly associated with cognitive impairment (OR = 1.147, 95% CI 1.082–1.223; Nagelkerke R² = 0.224), locomotor limitation (OR = 1.099, 95% CI 1.047–1.157; Nagelkerke R² = 0.227), hearing impairment (OR = 1.106, 95% CI 1.056–1.164; Nagelkerke R² = 0.191), and visual impairment (OR = 1.161, 95% CI 1.054–1.290; Nagelkerke R² = 0.199), but not with nutritional risk (Nagelkerke R² = 0.045) or psychological impairment (Nagelkerke R² = 0.022), suggesting that these domains may be driven by factors beyond chronological age and comorbidity burden. To examine whether age modified the association between CCI and IC, a multiplicative interaction term (CCI × age) was introduced in the regression model. The interaction term did not reach statistical significance (β = −0.009, 95% CI − 0.019 to 0.001, *p* = 0.067), although the estimate was suggestive of a non-significant trend towards effect modification by age. In age-stratified analyses (< 70 years, *n* = 105; ≥70 years, *n* = 95), CCI was not independently associated with IC in either stratum (younger: β = +0.028, 95% CI − 0.104 to 0.159, *p* = 0.676; older: β = −0.079, 95% CI − 0.250 to 0.091, *p* = 0.357), while age remained associated in both groups (all *p* < 0.001). When analyses were further stratified by 5-year age bands, a trend towards a stronger negative association between CCI and IC was observed in women older than 75 years (β = −0.235, 95% CI − 0.478 to 0.008, *p* = 0.057; unadjusted model), although this did not reach statistical significance, likely reflecting limited statistical power within this subgroup (*n* = 43). In additional age-stratified analyses, neither years since menopause nor reproductive lifespan showed significant associations with IC in either younger or older women (younger: β = −0.003, *p* = 0.857 and β = −0.001, *p* = 0.953, respectively; older: β = −0.013, *p* = 0.537 and β = −0.003, *p* = 0.894, respectively), suggesting that reproductive history does not independently influence IC even in the early post-menopausal phase.

## Discussion

In this cross‑sectional study of postmenopausal women attending a tertiary outpatient clinic, impairment in IC was highly prevalent across all the WHO‑defined domains, with sensory, psychological, and locomotor components most frequently affected. Although comorbidity burden, as measured by the CCI, was inversely associated with preserved IC in the unadjusted analyses, this association was no longer evident after accounting for age. Age was significantly and independently associated with IC, explaining a substantial proportion of its variability, whereas comorbidity burden appeared largely age‑dependent.

The high prevalence of IC impairment observed in this cohort underscores the vulnerability of older postmenopausal women with osteoporosis or osteopenia, even in the absence of severe disability. Sensory impairment, particularly visual limitation, was almost ubiquitous. This finding should be interpreted cautiously, as the WHO 6/12 threshold is intentionally sensitive and designed for early detection rather than clinical diagnosis. Nevertheless, the near-universal identification of visual impairment highlights the potential yield of systematic sensory screening in outpatient populations and aligns with the ICOPE framework’s emphasis on early identification of functional decline. Psychological impairment and locomotor limitation were also common, reinforcing the multidimensional nature of IC and the need for integrated, multidomain assessment in routine clinical practice.

The inverse correlation between comorbidity burden and IC components confirms that multimorbidity and functional reserves are closely linked constructs [17, 18]. However, the attenuation of this association after adjustment for age suggests that chronological ageing is the primary driver underlying declines in IC, with comorbidity acting largely as a correlate rather than an independent determinant. This finding supports the conceptual distinction, central to the WHO healthy ageing framework, between disease burden and intrinsic capacity. While chronic conditions accumulate with age and contribute to functional decline, IC appears to capture broader age-related physiological changes that are not fully explained by diagnosed morbidity alone.

Longitudinal evidence further supports this interpretation. A systematic review and meta-analysis by Sánchez et al. [6] showed that declines in IC are associated with an increased risk of subsequent disability in both basic and instrumental activities of daily living (BADL and IADL), as well as with higher mortality, independently of disease status. Consistently, Campbell et al. [17], analysing data from the English Longitudinal Study of Ageing, reported that IC predicted disability, hospitalisation, and mortality after adjustment for multimorbidity. In a large cohort of community-dwelling older adults in China, Zhao et al. [18] found that IC predicted ADL disability more accurately than multimorbidity status, further indicating that functional reserve captures prognostic information beyond disease burden.

Thus, reliance on comorbidity burden alone may fail to identify individuals with substantial functional impairment but relatively modest disease burden, particularly in domains such as sensory and psychological capacity, which are not captured by the CCI, but are nonetheless associated with adverse outcomes, including falls, fracture risk, and reduced quality of life [19]. Conversely, the accounting of the chronic disease burden remains clinically relevant for disease management and prognostication.

Notably, in our study, age accounted for nearly one-third of the variability in preserved IC components and demonstrated a stronger association with IC than comorbidity burden in correlation analyses. From a clinical perspective, this reinforces the value of IC as a complementary construct to multimorbidity in the assessment of older adults, particularly in populations where chronic conditions are highly prevalent and provide limited discriminatory power [19, 20]. The absence of independent associations between IC and reproductive or socioeconomic factors after age adjustment warrants consideration. Although reproductive lifespan and years since menopause have been variably linked to later-life health outcomes, their lack of association with IC in this cohort suggests that, beyond chronological ageing, these factors may exert limited influence on current functional reserves in postmenopausal women attending specialist care. Similarly, the lack of independent effects of education and occupational status may reflect reduced socioeconomic heterogeneity or the overriding influence of age and clinical status in this tertiary population. Several limitations of this study should be acknowledged. The cross-sectional design precludes causal inference and does not capture trajectories of intrinsic capacity over time. The use of screening tools aligned with the ICOPE framework, while appropriate for identifying early impairment, may overestimate domain-specific deficits, particularly in sensory function. In addition, the study population was restricted to women receiving specialist care for bone health, which may limit generalisability to community-dwelling populations or to men. Nonetheless, the consecutive recruitment strategy and comprehensive multidomain assessment strengthen the internal validity of the findings. Furthermore, while domain-specific analyses were performed, the exploration of specific combinations of IC domain impairments was beyond the scope of the present study given the sample size constraints and represents an avenue for future investigation.

In conclusion, this study demonstrates that IC is markedly compromised in older postmenopausal women with bone disorders and that age, rather than comorbidity burden, is strongly associated with functional reserves. These findings support the conceptual value of IC as a construct distinct from multimorbidity and reinforce the importance of age-sensitive, multidimensional functional assessment in clinical practice. Concurrent assessment of IC and comorbidity may offer a more complete characterisation of vulnerability of older adults, supporting the incorporation of ICOPE-based IC screening alongside standard comorbidity scoring in comprehensive, person-centred geriatric evaluation. Longitudinal studies are needed to clarify the temporal relationships between ageing, comorbidity accumulation, and trajectories of IC, and to identify opportunities for early, targeted interventions to preserve functional ability in later life.

## Supplementary Information

Below is the link to the electronic supplementary material.

Supplementary Material 1

## Data Availability

The datasets generated and/or analysed during the current study are available from the corresponding author on reasonable request.
