# Supplementary material for: Intrinsic capacity and comorbidity burden capture distinct but age-driven dimensions of health in older postmenopausal women
Source: Aging Clin Exp Res. 2026 Jul 11;38(1):203. doi: 10.1007/s40520-026-03456-7 (PMC13645857; doi:10.1007/s40520-026-03456-7)
Supplement: Supplementary file 1 [file 40520_2026_3456_MOESM1_ESM.docx]

**Supplementary Table 1.** Distribution of Charlson Comorbidity Index (CCI) scores according to the number of preserved intrinsic capacity (IC) components (n = 200).

| Preserved IC components | n (%) | CCI mean ± SD | CCI median (IQR) |
| --- | --- | --- | --- |
| 0 | 4 (2.0) | 6.50 ± 2.65 | 6.0 (4.75–7.75) |
| 1 | 14 (7.0) | 4.64 ± 1.22 | 5.0 (4.00–5.00) |
| 2 | 28 (14.0) | 4.04 ± 1.43 | 3.5 (3.00–5.00) |
| 3 | 50 (25) | 3.40 ± 2.02 | 3.0 (2.00–4.00) |
| 4 | 58 (29.0) | 3.43 ± 1.77 | 3.0 (2.00–4.00) |
| 5 | 41 (20.5) | 2.80 ± 1.49 | 3.0 (2.00–3.00) |
| 6 | 5 (2.5) | 1.40 ± 2.07 | 1.0 (0.00–1.00) |

***Abbreviations****:IQR, interquartile range; IC, intrinsic capacity; SD, standard deviation.*

**Supplementary Table 2.** Association between reproductive and socioeconomic variables and preserved intrinsic capacity (IC) components: unadjusted (Spearman's ρ) and age-adjusted multivariable linear regression analyses.

| Variable | Spearman's ρ | p (unadjusted) | β (age-adjusted) | 95% CI | p (age-adjusted) |
| --- | --- | --- | --- | --- | --- |
| **Reproductive factors** |  |  |  |  |  |
| Years since menopause | −0.396 | <0.001 | −0.009 | −0.033 to 0.014 | 0.433 |
| Reproductive lifespan (yr) | −0.042 | 0.569 | 0.000 | −0.032 to 0.032 | 0.995 |
| **Socioeconomic factors** |  |  |  |  |  |
| Education level | 0.165 | 0.020 | 0.140 | −0.035 to 0.315 | 0.116 |
| Occupational status | −0.024 | 0.732 | 0.044 | −0.113 to 0.200 | 0.583 |

Education level coded as: 1 = primary school, 2 = middle school, 3 = high school, 4 = university degree. Occupational status coded as: 1 = housewife, 2 = employed, 3 = unemployed, 4 = retired. IC, intrinsic capacity; CI, confidence interval.
